# Do birth outcomes mediate the association between drug use in pregnancy and neonatal metabolic bone disease? A prospective cohort study of 10,801 Chinese women

**DOI:** 10.3389/fpubh.2024.1377070

**Published:** 2024-09-30

**Authors:** Honglin Jiang, Jialin Guo, Jing Li, Chunlin Li, Wenchong Du, Federico Canavese, Feng Xie, Huajing Li, Jian Yang, Hao Ying, Jing Hua

**Affiliations:** ^1^Shanghai First Maternity and Infant Hospital, Tongji University School of Medicine, Shanghai, China; ^2^NTU Psychology, Nottingham Trent University, Nottingham, United Kingdom; ^3^CHU Lille, Department of Pediatric Orthopedic Surgery, Lille University, Lille, France; ^4^Department of Orthopedics and Sports Medicine, Shanghai United Family Hospital, Shanghai, China; ^5^Aigora Technology PTE. LTD, TRIVEX, Singapore, Singapore

**Keywords:** metabolic bone disease, medication during pregnancy, birth outcome, prematurity, low birth weight, small for gestational age

## Abstract

**Background:**

Prenatal drug use may cause toxicity to bone health in newborns. We aimed to examine whether birth outcomes mediate the association between medication use and neonatal metabolic bone disease (MBD).

**Methods:**

A prospective cohort of 10,801 pregnant women (17–49 years) and their infants followed at a single center from 1 January 2012 to 31 December 2021 were included. Based on four single drugs, comprehensive medication use was determined and categorized into three groups using latent-class analysis: group 1 included antibiotics and furosemide or less than two drugs except for MgSO_4_; group 2 included MgSO_4_ without antibiotics or furosemide; and group 3 encompassed dexamethasone and antibiotics. Mediation analysis was conducted to assess the mediating effects of prematurity, low birth weight (LBW), and small for gestational age (SGA).

**Results:**

There were 138 (1.3%) infants with MBD; 2,701 (25%) were born preterm, 1717 (15.9%) had LBW, and 303 (2.8%) were SGA. Pregnant women in groups 2 and 3 were 2.52 to 14.66 times more likely to deliver an infant with MBD than those in group 1. Only LBW showed a significant mediating effect on the association between comprehensive medication use and MBD, with a mediation proportion of 51.8% (45.0–64.1%, *p* < 0.001).

**Conclusion:**

Comprehensive medication use during pregnancy was associated with an increased risk of neonatal MBD, largely mediated by LBW. Early antepartum monitoring and prevention targeting adverse birth outcomes are necessary to mitigate the risk of MBD.

## Introduction

Metabolic bone disease (MBD) remains a common comorbidity in infants, particularly those who are born preterm, have low birth weight (LBW), are small for gestational age (SGA), or have chronic conditions, despite notable advancements in neonatal care (1–3). Among the various factors contributing to MBD, birth weight has been identified as the most influential independent risk factor (4, 5). The improved survival rate of LBW infants has coincided with an increased incidence of MBD, which reaches 32 and 54% in very low birth weight (VLBW, < 1,500 g) and extremely low birth weight (ELBW, < 1,000 g) infants, respectively (6). LBW and SGA infants often experience placental insufficiency, resulting in decreased active mineral transport during gestation, leading to inadequate mineral supply, negatively affecting bone development and potentially resulting in senile osteoporosis in the long term (7).

Pharmacological treatments during pregnancy are widespread, and concerns regarding their fetal safety have garnered increased attention. Epidemiological investigations have revealed that various medications can lead to different adverse neonatal outcomes, albeit with inconsistent findings across different drug categories (8–10). For example, magnesium sulfate (MgSO_4_), commonly used in preterm deliveries, has been linked to reduced calcium levels and bone problems in newborns and developing fetuses, particularly when used for extended periods of 5–7 days during pregnancy (11); diuretic therapy (e.g., furosemide) has been associated with increased renal calcium loss and skeletal abnormalities in newborns (4). Animal experiments have also demonstrated that prenatal administration of certain drugs, such as antibiotics and synthetic glucocorticoids (e.g., dexamethasone), results in osteo-cartilaginous developmental toxicity in the offspring (12).

Existing studies primarily focus on the effects of individual drugs (11, 13). However, given the heterogeneous nature of pharmacokinetic mechanisms, it becomes challenging for clinicians to evaluate the overall impact of multiple drugs administered during pregnancy on the risk of MBD development and to propose rational pharmacological therapies (14, 15). Additionally, limited research has investigated the intricate relationships between birth outcomes, drug usage during pregnancy, and the occurrence of MBD in newborns (16–18).

In this study, we leveraged data from a large cohort of pregnant women in Shanghai, China, with a follow-up period of 10 years. We constructed a comprehensive variable that encompassed the use of different types of drugs during pregnancy and assessed its association with neonatal MBD, considering the mediating effect of birth outcomes.

## Methods

### Study population

A cohort of pregnant women (singleton pregnancy) in the first and/or second trimester of pregnancy was recruited from 1 January 2012 at the First Maternal and Infant Hospital in Shanghai. All data were prospectively collected, and the maternal information and newborn birth outcomes database were updated until 31 December 2021. Women with multiple pregnancies (*n* = 2,559) and those with missing information (*n* = 3,158) were excluded from the study. A previous publication provides detailed information regarding the study design, data collection, and analysis of potential implications of missing data (19).

In total, the current analysis included 10,801 women aged 17–49 years at enrollment and their respective infants. A diagnosis of MBD in infants was established based on a peak serum alkaline phosphatase level exceeding 500 U/L 72 h after birth. The study was approved by the ethics committees of Shanghai First Maternity and Infant Hospital and Tongji University School of Medicine (No. KS21251). All participants provided written or oral consent for data collection and analysis prior to their inclusion in the study. The data included in the analysis were fully anonymized.

### Assessment of medication use in pregnancy

Four commonly prescribed medications during any stage of pregnancy were recorded:

1. *MgSO_4_*, to treat preterm labor via injection before anticipated early preterm delivery (20) or to prevent and control seizures in preeclampsia and eclampsia (21);
2. *Furosemide*, to treat hypertensive disorders (22) or edematous states (23) orally or intravenously;
3. *Dexamethasone*, to accelerate the maturation of fetal lungs, which is given to women at risk of preterm birth or before planned preterm birth, typically as two injections (24);
4. *Antibiotics*, to treat maternal infections including urinary and respiratory tract infections when necessary (25).

### Creation of a comprehensive medication use variable

To measure the overall effect of medication use during pregnancy, a comprehensive medication use variable was created based on the four drugs using latent-class analysis with the R package ‘poLCA’ (26). Latent-class analysis allows for the generation of an unmeasured latent variable consisting of mutually exclusive latent groups based on a set of categorical observed variables (27).

Three latent classes were identified as group 1, group 2, and group 3 of medication use during pregnancy based on item–response probabilities. Group 1 included participants who took fewer than two types of drugs (except for MgSO_4_) or received antibiotics and furosemide during pregnancy. Group 2 comprised participants who definitely took MgSO_4_ but not in combination with antibiotics or furosemide. Group 3 encompassed participants with a history of dexamethasone and antibiotic use, with or without the other two drugs (including all four medications).

Further details of the latent-class analysis can be found in Supplementary methods.

### Definition of birth outcomes

Prematurity, birth weight, and SGA are interrelated factors associated with gestational age. Neonates with a gestational age of less than 37 weeks and greater than or equal to 28 weeks are classified as born preterm. A neonatal birth weight of less than 2,500 g is considered as LBW. SGA is defined as the birth weight below the 10th percentile at the corresponding gestational age. To facilitate the identification and interpretation of the model, these potential mediating factors were converted into categorical variables. A value of 1 was assigned to indicate the presence of adverse events, including preterm birth, LBW, and SGA, whereas a value of 0 indicated their absence.

### Statistical analysis

Initial continuous variables were first transformed into dummy or categorical variables, including age at pregnancy (≤ 20, 21–30, 31–40, and > 40 years), pre-pregnancy body mass index (BMI; < 18.5, 18.5–23.9, 24–27.9, and ≥ 28), and the latent class of medication use (group 1, group 2, and group 3). Baseline characteristics and exposures during pregnancy were described as numbers and percentages across the different groups of comprehensive medication use. Differences between these groups were tested using the chi-square test or Fisher’s exact test.

The modified Poisson regression model was used to estimate the risk ratio (RR) and 95% confidence interval (CI) of MBD outcomes associated with comprehensive medication use in pregnancy and each birth outcome. We constructed two models in each step of the analysis to adjust for potential confounders [i.e., factors that were significantly associated with MBD outcomes (19)]. In addition to medication use and hypothesized mediators, model 1 also included age at pregnancy, pre-pregnancy BMI, employment status, and parity for adjustment, while model 2 additionally adjusted for folic acid deficiency, vitamin D deficiency, iron supplementation, calcium supplementation, placenta previa, placental abruption, gestational hypertension, and fever.

A parallel multiple mediator model was built to estimate the mediating role of the birth outcomes of interest in the occurrence of MBD (Supplementary Figure S1). Estimates of the direct, indirect, and total effects of medication during pregnancy on MBD were calculated from two models with corresponding adjusted variables, respectively. The proportion of mediation by mediators (three birth outcomes) for the association between comprehensive medication use and MBD was defined as the proportion of the indirect effect in the total effect. Quasi-Bayesian approximation method was used to test the significance of the mediating effect of each mediator with 50 simulations. Bonferroni correction was used to control for type I error for multiple hypothesis tests in the multiple mediator model.

We also conducted a stratified analysis by latent class of comprehensive medication use to investigate the associations of birth outcomes with MBD events among pregnant women with different subgroups of medication use.

We conducted sensitivity analyses by (1) repeating all analyses with each single medication use in pregnancy—that is, dexamethasone, MgSO_4_, antibiotics, and furosemide; and (2) introducing exposure (medication use)–mediator (PTB and LBW) interactions in the mediation models to explore causal assumptions and validate the robustness of the findings.

All analyses were performed using R version 4.4.1. A two-sided *p*-value <0.05 was considered to be significant for comparisons.

## Results

### Characteristics of the participants

Table 1 shows the baseline characteristics of the 10,801 pregnant women included in the study with a mean age of 29.7 ± 3.9 years. After birth, 138 (1.3%) infants were diagnosed with MBD, 2701 (25.0%) were preterm, 1717 (15.9%) had LBW, and 303 (2.8%) had SGA.

**Table 1:** Baseline characteristics of participants according to latent class of comprehensive medication use during pregnancy.

	Category of comprehensive medication use			
	Total; *n* = 10,801 (%)	Group 1; *n* = 8,975 (%)	Group 2; *n* = 870 (%)	Group 3; *n* = 956 (%)
Gave birth to MBD infants	138 (1.3)	39 (0.4)	50 (5.7)	49 (5.1)
Age at pregnancy (y)^*^				
≤ 20	50 (0.5)	45 (0.5)	4 (0.5)	1 (0.1)
21–30	6,890 (63.8)	5,908 (65.8)	481 (55.3)	501 (52.4)
31–40	3,780 (35.0)	2,967 (33.1)	373 (42.9)	440 (46.0)
> 40	81 (0.7)	55 (0.6)	12 (1.4)	14 (1.5)
Pre-pregnancy BMI^*^				
< 18.5	1,368 (12.7)	1,194 (13.3)	80 (9.2)	94 (9.8)
18.5–23.9	7,452 (69.0)	6,258 (69.7)	561 (64.5)	633 (66.2)
24–27.9	1,444 (13.3)	1,144 (12.7)	149 (17.1)	151 (15.8)
≥ 28	537 (5.0)	379 (4.2)	80 (9.2)	78 (8.2)
Han ethnicity	10,600 (98.1)	8,809 (98.2)	853 (98.0)	938 (98.1)
Employment^*^	9,363 (86.7)	7,837 (87.3)	714 (82.1)	812 (84.9)
Health insurance^*^	8,582 (79.5)	7,156 (79.7)	655 (75.3)	771 (80.6)
Parity (= 1)^*^	8,810 (81.6)	7,383 (82.3)	705 (81.0)	722 (75.5)
Uterine scarring^*^	1,001 (9.3)	786 (8.8)	74 (8.5)	141 (14.7)
Folic acid deficiency	765 (7.1)	635 (7.1)	63 (7.2)	67 (7.0)
Ferritin deficiency	992 (9.2)	803 (8.9)	92 (10.6)	97 (10.1)
Vitamin D deficiency^*^	2,575 (23.8)	2,117 (23.6)	199 (22.9)	259 (27.1)
Gestational anemia	1,155 (10.7)	978 (10.9)	75 (8.6)	102 (10.7)
Folic acid supplementation^*^	645 (6.0)	503 (5.6)	49 (5.6)	93 (9.7)
Iron supplementation^*^	3,949 (36.6)	3,196 (35.6)	289 (33.2)	464 (48.5)
Calcium supplementation^*^	1,013 (9.4)	797 (8.9)	102 (11.7)	114 (11.9)
Vitamin D supplementation^*^	2,742 (25.4)	2,228 (24.8)	239 (27.5)	275 (28.8)
Placenta previa^*^	249 (2.3)	132 (1.5)	35 (4.0)	82 (8.6)
Placental abruption^*^	165 (1.5)	105 (1.2)	30 (3.4)	30 (3.1)
Gestational diabetes^*^	1,439 (13.3)	1,126 (12.5)	160 (18.4)	153 (16.0)
Gestational hypertension^*^	999 (9.2)	362 (4.0)	459 (52.8)	178 (18.6)
Renal disease	44 (0.4)	31 (0.3)	6 (0.7)	7 (0.7)
Fever^*^	1,577 (14.6)	1,414 (15.8)	74 (8.5)	89 (9.3)

MBD, metabolic bone disease; BMI, body mass index. Values are presented as numbers and percentages. Characteristics were compared between different groups of comprehensive medication use. *p*-values were calculated using the chi-square test or Fisher’s exact test for categorical variables. *Significant differences existed between groups (*p* < 0.05).

As for the comprehensive use of drugs during pregnancy, 8,975 (83.1%) of participants were classified in group, 1,870 (8.1%) in group 2, and 956 (8.8%) in group 3. Among the three groups, group 1 had a lower likelihood of delivering an infant with MBD and a lower prevalence of gestational complications and comorbidities, except for fever. In contrast, group 2 appeared to be more likely to deliver a child with MBD, with a higher BMI, no health insurance, and a higher prevalence of placental abruption, gestational diabetes, and hypertension. Participants in group 3 tended to be older, unemployed, and multiparous, with uterine scarring, vitamin D deficiency, and nutrient supplementation during pregnancy.

### Mediation analysis of birth outcomes on associations of medication use during pregnancy with MBD

After adjusting for maternal demographics in model 1, including age at pregnancy, pre-pregnancy BMI, employment status, and parity, the RRs when participants in group 2 and group 3 were compared to those in group 1 were 3.37 (95% CI 3.14–3.62) and 3.58 (95% CI 3.35–3.82) for prematurity, 5.38 (95% CI 4.89–5.91) and 5.30 (95% CI 4.73–5.94) for birth weight, and 4.82 (95% CI 3.68–6.31) and 3.77 (95% CI 2.84–4.99) for SGA, respectively. The RRs for the three birth outcomes were similar in model 2 (including covariates of maternal demographics, prenatal nutritional conditions, and gestational complications/comorbidities; Supplementary Table S1).

Regarding comprehensive drug use, pregnant women in group 2 and group 3 had a 2.52- to 14.66-fold increased risk of delivering an infant with MBD compared to those in group 1, after adjustment for potential mediating factors and other covariates in model 1 and model 2.

The RRs without adjustment for birth outcomes were larger than those adjusted for prematurity and LBW but similar to those adjusted for SGA. Higher risks for MBD were observed in preterm infants [63.99 (95% CI 28.31–144.63) in model 1; 60.92 (95% CI 26.70–138.99) in model 2] and infants with LBW [95% CI 75.23 (38.53–146.89) in model 1; 74.69 (95% CI 38.03–146.71) in model 2]. SGA was not associated with MBD in either model, indicating that SGA did not mediate the association between drug use during pregnancy and the occurrence of MBD (Table 2). Thus, SGA was not selected as a mediating factor in the subsequent analyses. The results of the subgroup analysis stratified by groups of comprehensive medication use remained similar to those of the main analyses (Supplementary Figure S2; Supplementary Table S2).

**Table 2:** Relative risks of MBD associated with comprehensive medication use during pregnancy and potential mediating factors.

Factors	MBD (RR, 95% CI)	
	Model 1^a^	Model 2^b^
Comprehensive medication use		
Group 2^c^		
Unadjusted	12.48 (8.13–19.14)^**^	14.64 (8.83–24.28)^**^
Adjusted for prematurity	4.08 (2.66–6.27)^**^	4.08 (2.66–6.25)^**^
Adjusted for LBW	2.73 (1.76–4.23)^**^	3.08 (1.92–4.93)^**^
Adjusted for SGA	12.58 (8.15–19.41)^**^	14.66 (8.81–24.38)^**^
Group 3^c^		
Unadjusted	11.25 (7.35–17.20)^**^	12.30 (7.96–19.00)^**^
Adjusted for prematurity	3.46 (2.25–5.33)^**^	3.46 (2.27–5.29)^**^
Adjusted for LBW	2.52 (1.62–3.91)^**^	2.85 (1.83–4.46)^**^
Adjusted for SGA	11.31 (7.38–17.33)^**^	12.32 (7.96–19.07)^**^
Prematurity	63.99 (28.31–144.63)^**^	60.92 (26.70–138.99)^**^
LBW	75.23 (38.53–146.89)^**^	74.69 (38.03–146.71)^**^
SGA	1.92 (0.90–4.10)	1.53 (0.72–3.25)

MBD, metabolic bone disease; LBW, low birth weight; SGA, small for gestational age; RR, relative risk; CI, confidence interval. RR was calculated using modified Poisson regression. ^*^p < 0.05, ^**^p < 0.001.

a Adjusted for maternal demographics.

b Adjusted for maternal demographics. prenatal nutritional conditions, and gestational complications/comorbidities.

c Compared to group 1.

As shown in Table 3, after adjusting for prematurity and LBW, the direct effect of comprehensive drug use during pregnancy on the occurrence of MBD remained significant, with effect estimates ranging from 0.008 to 0.014 in both models (all *p* < 0.001). The proportion mediated by prematurity was 36.0% (4.2–50.4%, *p* = 0.040) in model 1, and 31.2% (3.0–47.7%, *p* = 0.040) in model 2, and the proportion mediated by LBW was 55.3% (47.4–65.4%, *p* < 0.001) in model 1, and 51.8% (45.0–64.1%, *p* < 0.001) in model 2; the mediating effect of prematurity was not considered significant after Bonferroni correction.

**Table 3:** Associations of comprehensive medication use during pregnancy with MBD incidence and mediating effects of prematurity and LBW.

Effects	Model 1^a^			Model 2^b^		
	Estimate	95% CI	*p*-value	Estimate	95% CI	*p*-value
**Prematurity**						
Total effect	0.013	0.006–0.017	< 0.001	0.015	0.007–0.019	< 0.001
Direct effect	0.008	0.005–0.013	< 0.001	0.010	0.006–0.016	< 0.001
Indirect effect	0.005	0.001–0.006	0.040	0.005	0.001–0.006	0.040
Mediation proportion (%)	36.0	4.2–50.4	0.040	31.2	3.0–47.7	0.040
**LBW**						
Total effect	0.025	0.016–0.052	< 0.001	0.028	0.017–0.054	< 0.001
Direct effect	0.011	0.006–0.021	< 0.001	0.014	0.007–0.025	< 0.001
Indirect effect	0.014	0.008–0.027	< 0.001	0.015	0.008–0.030	< 0.001
Mediation proportion (%)	55.3	47.4–65.4	< 0.001	51.8	45.0–64.1	< 0.001

MBD, metabolic bone disease; LBW, low birth weight; CI, confidence interval. A *p*-value less than 0.0083 is considered significant after the Bonferroni correction.

a Adjusted for maternal demographics.

b Adjusted for maternal demographics. prenatal nutritional conditions, and gestational complications/comorbidities.

In sensitivity analyses by single medication use during pregnancy, all drugs except antibiotics were associated with higher incidence rates of prematurity, LBW, and SGA (all *p* < 0.05, Supplementary Table S3). The results of the associations of dexamethasone and MgSO_4_ with incident MBD were consistent with comprehensive medication use, while furosemide was not significantly associated with MBD after adjusting for prematurity and LBW. We did not observe any association between antibiotic intake and the risk of MBD with or without adjustment for other covariates (Supplementary Table S4). Further mediation analyses showed that the proportion of the association between individual drugs used during pregnancy and neonatal MBD mediated by LBW ranged from 38.3% for MgSO_4_ to 66.8% for dexamethasone in the two models (Supplementary Table S5). When considering the medication–mediator interaction in the mediation models, LBW still showed a significant partial mediating effect (Supplementary Table S6).

## Discussion

In this study of a large cohort of 10,801 pregnant women (singleton pregnancy) from China, we observed an association between comprehensive medication use during pregnancy and the risk of neonatal MBD, both before and after considering variables related to birth outcomes (i.e., prematurity, LBW, and SGA). Our findings revealed that 51.8 to 55.3% could be attributed to LBW. Interestingly, neither prematurity nor SGA showed an association with the risk of MBD or had a mediating effect on the relationship between drug use and MBD. These results provide new insights into the potential mechanisms by which drug treatment during pregnancy may induce neonatal MBD.

Pregnant women often receive various medications, with more than 80% reporting the use of at least one medication during pregnancy (28). Several drugs have been demonstrated to have multi-organ developmental toxicity and the potential to interfere with fetal growth, leading to birth defects, prematurity, LBW, and other adverse pregnancy outcomes (12), as also evidenced in our study.

The four medications chosen in this study are commonly used in clinical practice for the prevention and management of associated complications in pregnant women and adverse neonatal outcomes. Specifically, our study revealed that the risk of MBD in offspring was higher when mothers used MgSO_4_ or a combination of dexamethasone and antibiotics at any point of pregnancy (groups 2 and 3) than those who received antibiotics and furosemide or less than two drugs excluding MgSO_4_ (group 1). These findings support the notion of fetal bone developmental toxicity resulting from maternal exposure to drugs and highlight the potential mechanism of drug–drug interactions (29). Consequently, the selection and dosage of medications during pregnancy require careful consideration.

Existing evidence has indicated a causal relationship between the prenatal use of drugs, such as synthetic glucocorticoids (30–32) and antibiotics (33–35), and impaired intrauterine bone development. For instance, animal models have shown that dexamethasone, a synthetic long-acting glucocorticoid commonly used to treat mothers at risk of preterm delivery, can delay fetal skeletal growth by inhibiting extracellular matrix synthesis and downregulating insulin-like growth factor 1 signaling (32). It has also been found to induce cartilage dysplasia after repeated exposure by inhibiting the cartilage transforming growth factor *β* signaling pathway (30). Antibiotics are widely used during pregnancy to manage common infections (25), and their prenatal use has been associated with cartilage damage and inhibition of bone growth across various antibiotic classes (33, 34). In particular, our study revealed that the combined use of dexamethasone and antibiotics during pregnancy carried a higher risk for MBD in infants than their individual use, suggesting that increased frequency of exposure to these medications during gestation may alter fetal bone formation and mineralization. In addition to intrinsic drug toxicity, considerations of drug–drug interactions (29, 36) and alterations in pharmacokinetics resulting from physiologic changes in pregnancy (14, 15, 37) require careful monitoring and assessment when prescribing medications to pregnant women.

Furthermore, our study demonstrated that maternal exposure to MgSO_4_ increased the risk of MBD, irrespective of the concurrent use of other medications, consistent with the previous reports (19). The U.S. Food and Drug Administration advised against prolonged use of MgSO_4_ to halt preterm labor due to bone changes observed in exposed infants, including osteopenia and fractures (38). Case reports and a recent meta-analysis suggested an association between long-term, high-dose exposure to antenatal MgSO_4_ for tocolysis and neonatal bone abnormalities (8, 39). This may be explained by the competition between calcium and magnesium ions on the bone surface (40).

The correlations between adverse birth outcomes and MBD have been extensively discussed in the literature (2, 5, 18, 41), and our study confirmed that prematurity and LBW are associated with an increased risk of MBD, regardless of medication use. Bone mineralization in premature infants is affected in the neonatal period primarily due to deficient mineral reserves in the fetus (2). The incidence of MBD increases with younger gestational age and lower birth weight (42, 43). The majority of preterm infants are either VLBW or ELBW and often experience inadequate mineral intake. This is due to difficulties in feeding during the early postnatal period because of illness or the need for long-term parenteral nutrition, which often does not provide sufficient mineral supply (2). However, we did not find a significant relationship between SGA and MBD, possibly due to the relatively smaller sample size of SGA infants, some of whom may have been constitutionally normal.

The selected medications are preventative measures taken before the actual event of unfavorable birth outcomes and the subsequent diagnosis of MBD, which supports the causal assumption. Based on our mediation analysis, the effect of medication use during pregnancy on the development of MBD may be significantly mediated by LBW, accounting for 51.8 to 55.3% of the association between comprehensive medication use and the development of MBD, after adjusting for potential confounders. This effect constituted partial mediation, as the direct effect of medication was not significant when accounting for the medication–LBW interaction in the model. This suggests that the influence of prenatal drugs on neonatal MBD may be mediated by more complex pathways or biological mechanisms. Prematurity, however, did not show a significant mediating effect in our study. This indicates that the increased risk of MBD in neonates due to maternal medication use is more likely caused by the influence of these drugs on fetal bone deposition, rather than by gestational age at birth. The substantial proportion of mediation underscores the critical role of LBW in increasing the risk of MBD and suggests that early surveillance and therapeutic interventions during pregnancy to prevent adverse birth outcomes may reduce the risk in neonates. However, additional measures are still required to prevent neonatal MBD, such as informed decision-making regarding dosing schedules and critical assessment of the risks of therapeutic failure and adverse effects.

## Strengths and limitations

To the best of our knowledge, this is the first study to investigate the interactions between birth outcomes, maternal medication use, and the risk of MBD. The main strengths of this study include its large sample size derived from a 10-year dynamic cohort in Shanghai, China, which provided adequate statistical power to estimate the mediating effects of multiple birth outcomes and conduct stratified analyses (19). Furthermore, we constructed a comprehensive medication variable to capture the overall drug use during pregnancy. This comprehensive index of medication use can assist physicians make informed clinical decisions and enable public health policymakers to design targeted prevention strategies for populations at high risk of MBD. Our mediation analysis provides essential insights and highlights areas for further research to explore the detailed mechanisms of the association between medication during pregnancy and neonatal MBD. We also conducted sensitivity analyses to ensure the robustness of our findings and provided additional evidence on the associations between individual drugs and unintended adverse neonatal outcomes.

However, we also acknowledge certain limitations. First, we only included four common medications based on our clinical database, and information on the dosage and duration of these drugs was not collected for this study. Future studies should consider incorporating a broader range of drugs that are commonly used during pregnancy (e.g., antiemetics, analgesics, antidiabetic medications, and antidepressants) and investigate their degree of exposure. It is important to note that we were unable to correlate the specific medication usage to their intended therapeutic indications, which could independently influence the outcomes, such as MgSO4 use for tocolysis potentially being linked to both prematurity and bone disease, regardless of other variables. In addition, factors like neonatal enteral nutrition and parenteral nutrition could serve as potential mediators to explore the mechanism of gestational drug use in the development of MBD. Second, while the evaluation of mediators precedes the diagnosis of MBD, the exact sequence of their occurrence relative to each other may not be strictly sequential. Understanding whether these conditions are overlapping or co-occurring requires further mechanistic studies, for which our exploratory analysis offers significant foundational clues. Third, although we controlled for key personal characteristics and gestational complications/comorbidities, residual confounding remains a possibility, and causal inference cannot be made due to the observational nature of our study. Factors such as occupational exposure to air pollutants or indoor air quality were not measured in our study, which could influence both the mediators and the outcomes. However, the results from sensitivity analyses indicated that the effect estimates were robust to unobserved confounding factors. Although this study was conducted in a large cohort from Shanghai, China, the findings may not be generalizable to other populations due to differences in healthcare systems, medication usage patterns, and genetic predisposition. Further studies in diverse populations are needed to confirm our findings and improve their generalizability.

## Conclusion

In conclusion, our study of a large cohort of pregnant women (singleton pregnancy) from China demonstrated an association between comprehensive medication use during pregnancy and the risk of neonatal MBD, with LBW largely mediating the association. Early antepartum monitoring and prevention strategies targeting adverse birth outcomes, along with appropriate dosing schedules for prenatal medications, are necessary to mitigate the risk of MBD. Furthermore, careful attention should be given to the concurrent administration of multiple drugs and alterations in maternal dosage, as these factors may impact fetal exposure. Future studies examining other prenatal medications or exploring additional adverse birth outcomes may further enhance safety recommendations.

## Data Availability

The data analyzed in this study is subject to the following licenses/restrictions: the data that support the findings of this study are available from the First Maternal and Infant Hospital in Shanghai, but restrictions apply to the availability of these data, which were used under license for the current study, and so are not publicly available. Data are however available from the authors upon reasonable request and with permission of the First Maternal and Infant Hospital in Shanghai. Requests to access these datasets should be directed to Jing Hua, Jinghua@tongji.edu.cn.
