# Supplementary material for: Do birth outcomes mediate the association between drug use in pregnancy and neonatal metabolic bone disease? A prospective cohort study of 10,801 Chinese women
Source: Front Public Health. 2024 Sep 30;12:1377070. doi: 10.3389/fpubh.2024.1377070 (PMC11472830; doi:10.3389/fpubh.2024.1377070)
Supplement: Supplementary file 1 [file Data_Sheet_1.docx]

**Additional files**

**Title:** Do birth outcomes mediate the association between drug use in pregnancy and neonatal metabolic bone disease? A prospective cohort study of 10,801 Chinese women

These additional files contain one method, two figures, and six tables.

Content

[Supplemental methods 2](#_Toc173352261)

[Assessment of comprehensive medication use using latent class analysis 2](#_Toc173352262)

[Evaluation of the characteristics of each latent class 3](#_Toc173352263)

[Supplemental Figures 5](#_Toc173352264)

[Fig. S1 The model structure of the association of medication use and MBD mediated via birth outcomes 5](#_Toc173352265)

[Fig. S2 Association of potential mediating factors (prematurity, LBW and SGA) with MBD stratified by groups of comprehensive medication use during pregnancy in two adjusted models 6](#_Toc173352266)

[Supplemental Tables 7](#_Toc173352267)

[Table S1 Relative risks of potential mediating factors (prematurity/LBW/SGA) associated with comprehensive medication use during pregnancy 7](#_Toc173352268)

[Table S2 Relative risks of MBD associated with mediating factors by latent classes: subgroup analysis 8](#_Toc173352269)

[Table S3 Relative risks of potential mediating factors (prematurity/LBW/SGA) associated with single medication use during pregnancy 9](#_Toc173352270)

[Table S4 Relative risks of MBD associated with single medication use during pregnancy and potential mediating factors 10](#_Toc173352271)

[Table S5 Associations of single medication use with MBD incidence and the mediation effect of prematurity/LBW: sensitivity analysis 1 11](#_Toc173352272)

[Table S6 Associations of comprehensive medication use and the exposure-mediator interaction with MBD incidence: sensitivity analysis 2 12](#_Toc173352273)

# Supplemental methods

## Assessment of comprehensive medication use using latent class analysis

The use of four common individual drugs during pregnancy were recorded in the study: Magnesium Sulfate (MgSO_4_), Furosemide, Dexamethasone, and antibiotics. For the study purpose, we used these four parameters to generate an overall parameter in the subsequent analyses. There were two levels for each parameter (whether taking this drug or not). Latent class analyses with 2 to 3 of latent classes were conducted to select a reasonable model. Akaike information criterion (AIC), Bayesian information criterion (BIC), likelihood ratio statistic G^2^ and χ^2^ were used for the model selection. Item-response probability was a posterior probability and was used for defining latent classes.

The following figure shows that AIC, BIC, G^2^, and χ^2^ all continued to decrease as more latent classes were added. Thus, the three-latent-class solution was considered as the best model.


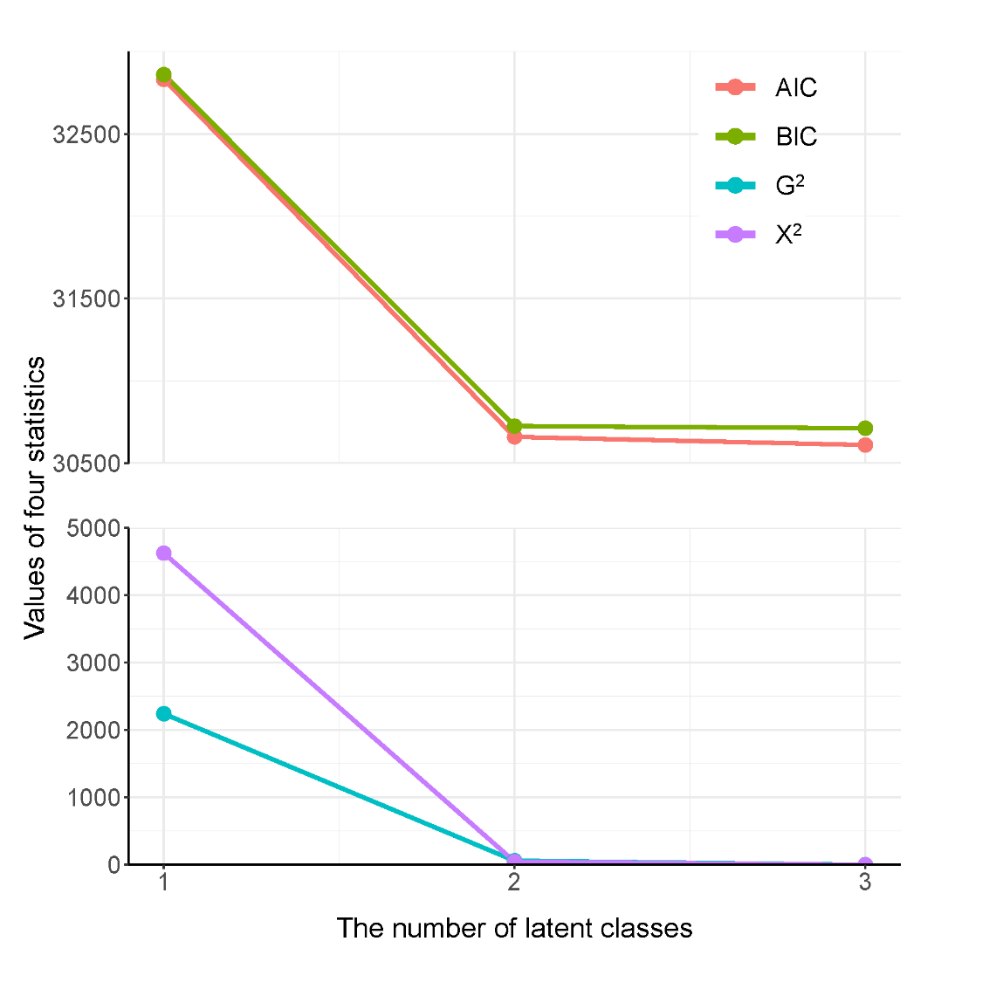


**Figure for supplemental methods.** Four statistics (AIC, BIC, G^2^, and χ^2^) in models with different numbers of latent classes.

## Evaluation of the characteristics of each latent class

For the three-latent-class solution, latent class 1 was characterized by not taking Dexamethasone, MgSO_4_, and Furosemide, which could be defined as Group 1; latent class 2 was characterized by taking MgSO_4_ and antibiotics, and not taking Furosemide and Dexamethasone, which could be defined as Group 2; latent class 3 was characterized by taking antibiotics, and not taking Furosemide, which could be defined as Group 3.

**Table for supplemental methods.** Item-response probabilities, population shares, and predicted class memberships of medication use in models with two and three latent classes.

| **Item** | **Latent class 1** | **Latent class 2** | **Latent class 3** |
| --- | --- | --- | --- |
| **Two-latent-class solution** |  |  |  |
| Dexamethasone 1 | **0.9874** | 0.4992 | NA |
| Dexamethasone 2 | 0.0126 | **0.5008** | NA |
| Magnesium sulfate 1 | **0.9764** | 0.4422 | NA |
| Magnesium sulfate 2 | 0.0236 | **0.5578** | NA |
| Antibiotics 1 | 0.4098 | 0.1440 | NA |
| Antibiotics 2 | **0.5902** | **0.8560** | NA |
| Furosemide 1 | **1.0000** | **0.8296** | NA |
| Furosemide 2 | 0.0000 | 0.1704 | NA |
| Estimated class population shares | 0.806 | 0.194 | NA |
| Predicted class memberships | 0.8276 | 0.1724 | NA |
| **Three-latent-class solution** |  |  |  |
| Dexamethasone 1 | **0.9715** | **0.6157** | **0.5062** |
| Dexamethasone 2 | 0.0285 | 0.3843 | 0.4938 |
| Magnesium sulfate 1 | **1.0000** | 0.0004 | 0.4865 |
| Magnesium sulfate 2 | 0.0000 | **0.9996** | **0.5135** |
| Antibiotics 1 | 0.4085 | 0.3812 | 0.0000 |
| Antibiotics 2 | **0.5915** | **0.6188** | **1.0000** |
| Furosemide 1 | **0.9985** | **0.9569** | **0.7557** |
| Furosemide 2 | 0.0015 | 0.0431 | 0.2443 |
| Estimated class population shares | 0.8151 | 0.0663 | 0.1186 |
| Predicted class memberships | 0.8227 | 0.0727 | 0.1046 |

Above all, we chose the three-latent-class solution and divided individuals into three groups of comprehensive medication use during pregnancy. In the three-latent class solution, the AIC is 30,611.18, and BIC is 30,713.20, G^2^ statistic is 1.16, and χ^2^ is 1.00. The practical definitions of which are shown in the following figure.


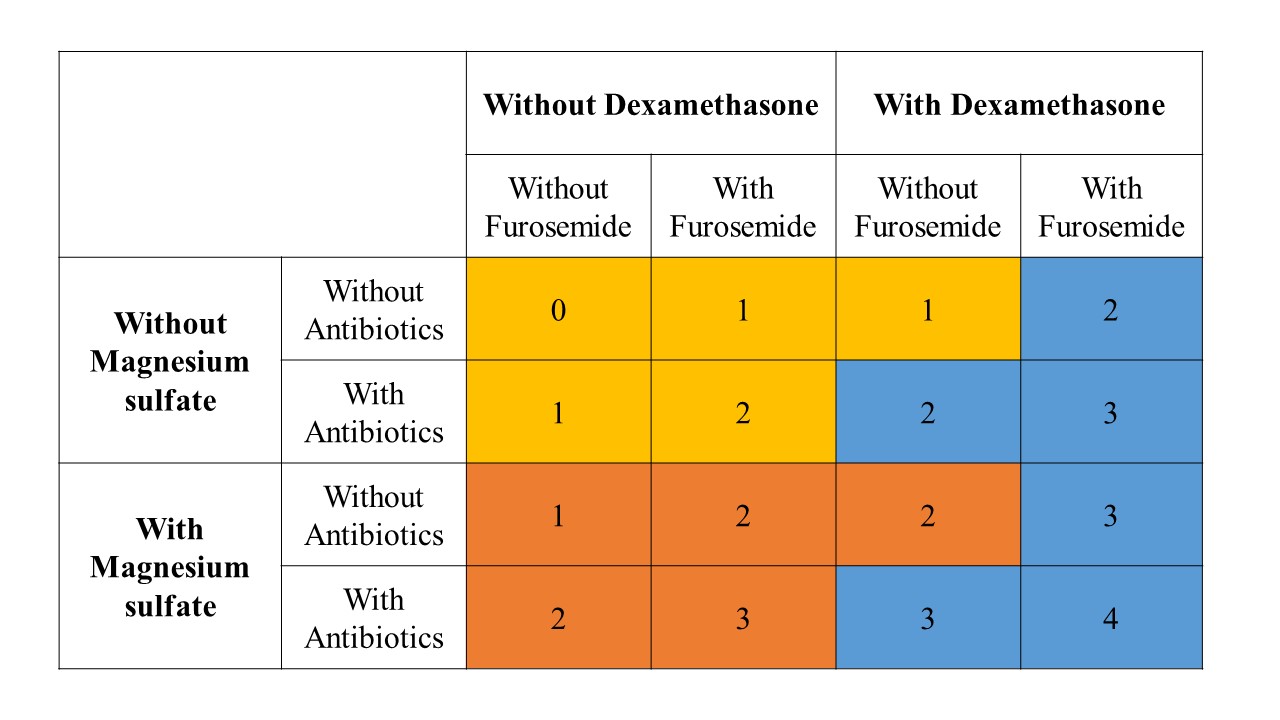


**Figure for supplemental methods.** Definitions of different groups of comprehensive medication use in the three-latent-class solution. The figure in each cell refers to the number of drug types used for pregnant women. The color in each cell represents the group of comprehensive medication use: 1) Yellow, Group 1; 2) Orange, Group 2; and 3) Blue, Group 3.

# Supplemental Figures


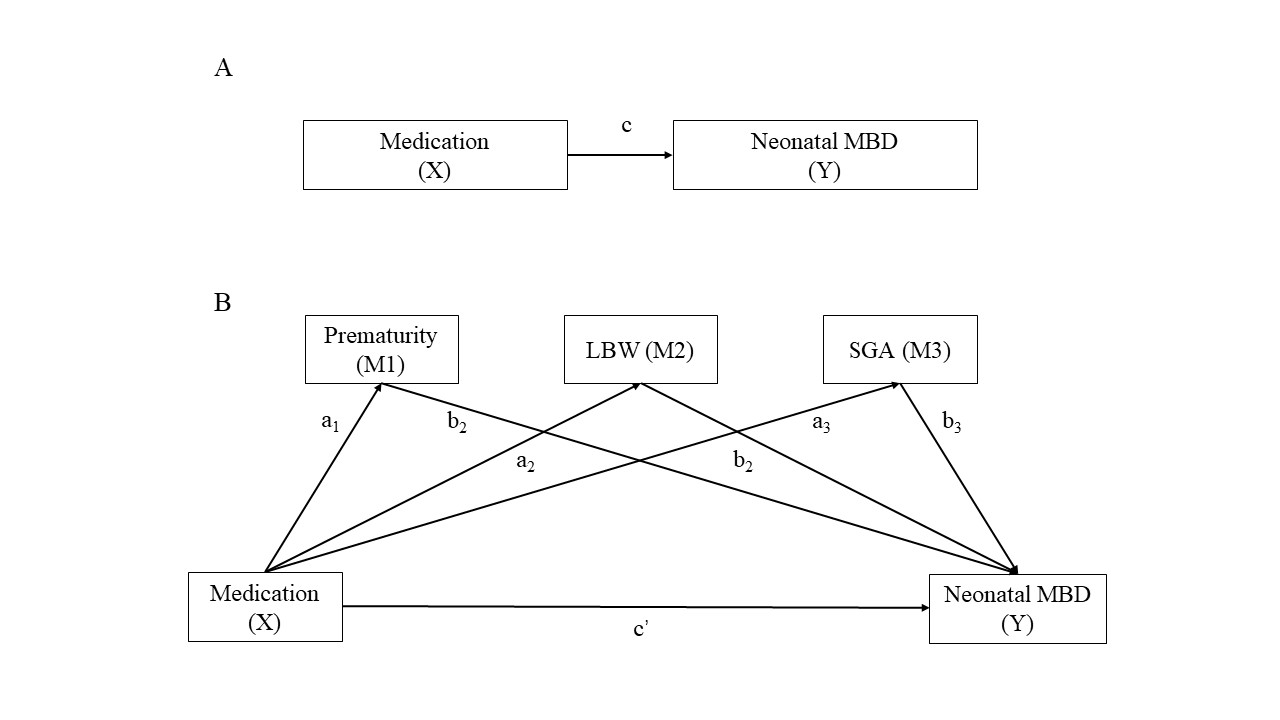


## Fig. S1 The model structure of the association of medication use and MBD mediated via birth outcomes

*X* refers to the independent variable (i.e., medication use during pregnancy), *Y* refers to the dependent variable (neonatal MBD), and *M* refers to mediation variables (“1” for prematurity, “2” for LBW and “3” for SGA). The total effect *c* of *X* on *Y* can be decomposed into direct effect *c'* and indirect effect *ɑ_1_b_1_+ɑ_2_b_2_+ɑ_3_b_3_*, in which *c'* is the effect of *X* on *Y* after controlling *M*, *a* is the effect of *X* on *M*, and *b* is the effect of *M* on *Y* after controlling *X*. A) The association of medication with MBD; B) the association of medication with MBD mediated by three potential mediators (prematurity, LBW, and SGA). MBD, metabolic bone disease; LBW, low birth weight; SGA, small for gestational age.


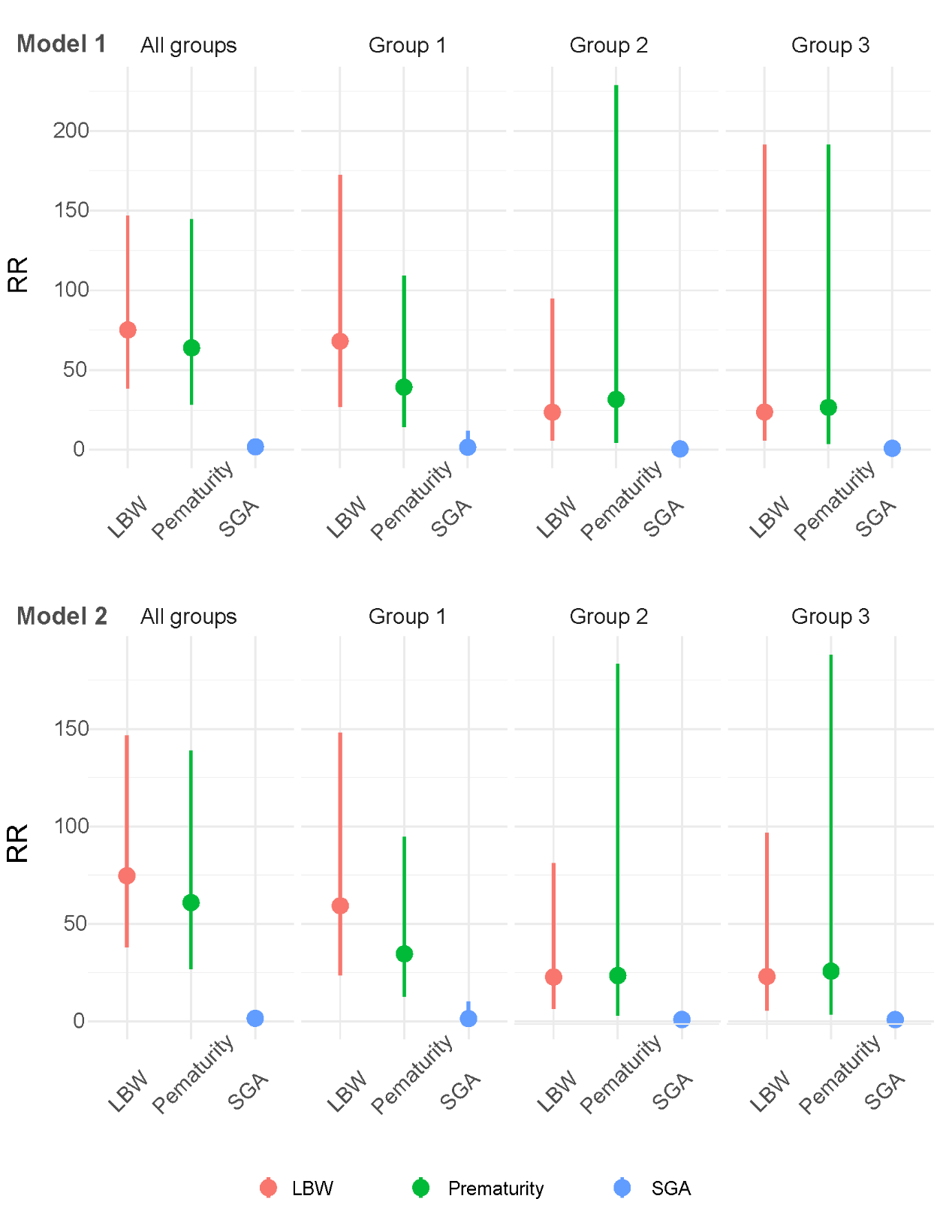


## Fig. S2 Association of potential mediating factors (prematurity, LBW and SGA) with MBD stratified by groups of comprehensive medication use during pregnancy in two adjusted models

Model 1, adjusted for maternal demographics. Model 2, adjusted for maternal demographics, prenatal nutritional conditions, and gestational complications/comorbidities. MBD, metabolic bone disease; LBW, low birth weight; SGA, small for gestational age; RR, relative risk.

# Supplemental Tables

## Table S1 Relative risks of potential mediating factors (prematurity/LBW/SGA) associated with comprehensive medication use during pregnancy

| **Medications** | **RR, 95% CI** | | |
| --- | --- | --- | --- |
|  | **Prematurity** | **LBW** | **SGA** |
| **Model 1^a^** | | | |
| **Comprehensive medication use** | | | |
| Group 1 | 1 (Reference) | 1 (Reference) | 1 (Reference) |
| Group 2 | 3.37 (3.14–3.62)^**^ | 5.38 (4.89–5.91)^**^ | 4.82 (3.68–6.31)^**^ |
| Group 3 | 3.58 (3.35–3.82)^**^ | 5.30 (4.73–5.94)^**^ | 3.77 (2.84–4.99)^**^ |
| **Model 2^b^** | | | |
| **Comprehensive medication use** | | | |
| Group 1 | 1 (Reference) | 1 (Reference) | 1 (Reference) |
| Group 2 | 3.18 (2.94–3.44)^**^ | 4.91 (4.39–5.49)^**^ | 2.62 (1.89–3.62)^**^ |
| Group 3 | 3.34 (3.11–3.59)^**^ | 5.15 (4.67–5.68)^**^ | 3.00 (2.24–4.02)^**^ |

LBW, low birth weight; SGA, small for gestational age; RR, relative risk; CI, confidence interval.

RR was calculated using modified Poisson regression.

^a^ Adjusted for maternal demographics.

^b^ Adjusted for maternal demographics, prenatal nutritional conditions, and gestational complications/comorbidities.

^*^*P* < 0.05, ^**^*P* < 0.001

## Table S2 Relative risks of MBD associated with mediating factors by latent classes: subgroup analysis

| **Groups** | **Model 1** | | |  | **Model 2** | | |
| --- | --- | --- | --- | --- | --- | --- | --- |
|  | **RR** | **LCI** | **UCI** |  | **RR** | **LCI** | **UCI** |
| Group 1 |  |  |  |  |  |  |  |
| Prematurity | 39.336^**^ | 14.168 | 109.210 |  | 34.597^**^ | 12.659 | 94.549 |
| LBW | 68.119^**^ | 26.912 | 172.421 |  | 59.265^**^ | 23.718 | 148.092 |
| SGA | 1.64 | 0.22 | 12.04 |  | 1.36 | 0.18 | 10.09 |
| Group 2 |  |  |  |  |  |  |  |
| Prematurity | 31.638^*^ | 4.379 | 228.573 |  | 23.479^*^ | 3.005 | 183.462 |
| LBW | 23.609^**^ | 5.875 | 94.880 |  | 22.724^**^ | 6.375 | 81.005 |
| SGA | 0.61 | 0.18 | 2.02 |  | 0.90 | 0.24 | 3.31 |
| Group 3 |  |  |  |  |  |  |  |
| Prematurity | 26.636^*^ | 3.709 | 191.266 |  | 25.733^*^ | 3.522 | 188.00 |
| LBW | 23.747^**^ | 5.833 | 96.673 |  | 23.042^**^ | 5.500 | 96.537 |
| SGA | 0.98 | 0.32 | 3.05 |  | 0.87 | 0.28 | 2.67 |

MBD, metabolic bone disease; LBW, low birth weight; SGA, small for gestational age; RR, relative risk; LCI, lower confidence interval; UCI, upper confidence interval.

RR was calculated using modified Poisson regression.

^*^*P* < 0.05, ^**^*P* < 0.001

## Table S3 Relative risks of potential mediating factors (prematurity/LBW/SGA) associated with single medication use during pregnancy

| **Medications** | **RR, 95% CI** | | |
| --- | --- | --- | --- |
|  | **Prematurity** | **LBW** | **SGA** |
| **Model 1^a^** | | | |
| **Single medication use** | | | |
| Dexamethasone | 3.24 (3.06–3.44)^**^ | 4.29 (3.96–4.65)^**^ | 2.51 (1.93–3.26)^**^ |
| Magnesium sulfate | 3.42 (3.23–3.62)^**^ | 5.31 (4.91–5.74)^**^ | 4.00 (3.17–5.05)^**^ |
| Antibiotics | 0.98 (0.91–1.05) | 1.02 (0.94–1.12) | 2.26 (1.71–2.98)^**^ |
| Furosemide | 1.92 (1.72–2.14)^**^ | 2.49 (2.18–2.85)^**^ | 3.73 (2.67–5.23)^**^ |
| **Model 2^b^** | | | |
| **Single medication use** | | | |
| Dexamethasone | 2.89 (2.71–3.07)^**^ | 3.84 (3.53–4.18)^**^ | 2.01 (1.55–2.62)^**^ |
| Magnesium sulfate | 3.27 (3.06–3.50)^**^ | 5.14 (4.69–5.63)^**^ | 2.26 (1.72–2.95)^**^ |
| Antibiotics | 0.96 (0.90–1.03) | 1.02 (0.93–1.11) | 2.18 (1.64–2.89)^**^ |
| Furosemide | 1.43 (1.28–1.60)^**^ | 1.68 (1.46–1.94)^**^ | 2.00 (1.34–2.97)^*^ |

LBW, low birth weight; SGA, small for gestational age; RR, relative risk; CI, confidence interval.

^a^ Adjusted for maternal demographics.

^b^ Adjusted for maternal demographics, prenatal nutritional conditions, and gestational complications/comorbidities.

^*^*P* < 0.05, ^**^*P* < 0.001

## Table S4 Relative risks of MBD associated with single medication use during pregnancy and potential mediating factors

| **Factors** | **MBD (RR, 95% CI)** | |
| --- | --- | --- |
|  | **Model 1^a^** | **Model 2^b^** |
| Dexamethasone |  |  |
| Unadjusted | 6.17 (4.39–8.66)^**^ | 5.80 (4.06–8.29)^**^ |
| Adjusted for prematurity | 2.04 (1.47–2.84)^**^ | 2.16 (1.55–3.00)^**^ |
| Adjusted for LBW | 1.58 (1.13–2.21)^*^ | 1.66 (1.19–2.32)^*^ |
| Adjusted for SGA | 6.09 (4.32–8.60)^**^ | 5.77 (4.02–8.28)^**^ |
| Magnesium sulfate |  |  |
| Unadjusted | 16.01 (10.97–23.38)^**^ | 20.11 (13.20–30.65)^**^ |
| Adjusted for prematurity | 5.17 (3.54–7.55)^**^ | 6.20 (4.13–9.32)^**^ |
| Adjusted for LBW | 3.53 (2.37–5.26)^**^ | 4.18 (2.73–6.41)^**^ |
| Adjusted for SGA | 16.18 (11.03–23.73)^**^ | 20.12 (13.18–30.71)^**^ |
| Antibiotics |  |  |
| Unadjusted | 0.79 (0.56–1.11) | 0.77 (0.55–1.10) |
| Adjusted for prematurity | 0.81 (0.58–1.13) | 0.82 (0.58–1.15) |
| Adjusted for LBW | 0.79 (0.57–1.10) | 0.81 (0.58–1.13) |
| Adjusted for SGA | 0.77 (0.55–1.09) | 0.76 (0.54–1.08) |
| Furosemide |  |  |
| Unadjusted | 3.03 (1.75–5.26)^**^ | 2.41 (1.35–4.32)^*^ |
| Adjusted for prematurity | 1.68 (0.98–2.88) | 1.60 (0.90–2.83) |
| Adjusted for LBW | 1.31 (0.76–2.25) | 1.39 (0.80–2.42) |
| Adjusted for SGA | 2.91 (1.65–5.12)^**^ | 2.35 (1.30–4.27)^*^ |

MBD, metabolic bone disease; LBW, low birth weight; SGA, small for gestational age; RR, relative risk; CI, confidence interval.

^a^ Adjusted for maternal demographics.

^b^ Adjusted for maternal demographics, prenatal nutritional conditions, and gestational complications/comorbidities.

^*^*P* < 0.05, ^**^*P* < 0.001

## Table S5 Associations of single medication use with MBD incidence and the mediation effect of prematurity/LBW: sensitivity analysis 1

| **Medications** | **Model 1^a^** | | | |  | **Model 2^b^** | | | |
| --- | --- | --- | --- | --- | --- | --- | --- | --- | --- |
|  | **Total effect** | **Direct effect** | **Indirect effect** | **Mediation proportion** |  | **Total effect** | **Direct effect** | **Indirect effect** | **Mediation proportion** |
| **Prematurity** | | | | | | | | | |
| Dexamethasone | 0.003 (0.002,0.004)^*^ | 0.002 (0.0003,0.003)^*^ | 0.001 (0.001,0.002) | 46.4 (16.4,86.1) |  | 0.003 (0.002,0.004)^*^ | 0.002 (0.001,0.003)^*^ | 0.001 (0.001,0.002) | 42.7 (14.0,75.7) |
| Magnesium sulfate | 0.003 (0.002,0.005)^*^ | 0.002 (0.001,0.005)^*^ | 0.001 (0,0.001) | 21.2 (0.1,36.8) |  | 0.003 (0.002,0.005)^*^ | 0.002 (0.001,0.005)^*^ | 0.001 (0-0.001) | 18.7 (-1.2,36.1) |
| Furosemide | 0.003 (-0.001,0.004) | 0.001 (-0.004,0.003) | 0.001 (0.001,0.002) | 53.2 (0,100) |  | 0.003 (-0.002,0.005) | 0.002 (-0.003,0.004) | 0.001 (0.0004,0.002) | 38.4 (0,100) |
| **LBW** | | | | | | | | | |
| Dexamethasone | 0.003 (0.003,0.006)^*^ | 0.001 (0.0002,0.002)^*^ | 0.002 (0.001,0.005)^*^ | 66.8 (47.3,92.7)^*^ |  | 0.004 (0.003,0.007)^*^ | 0.001 (0.0004,0.002)^*^ | 0.002 (0.002,0.005)^*^ | 63.7 (46.9,88.0)^*^ |
| Magnesium sulfate | 0.002 (0.002,0.004)^*^ | 0.001 (0.001,0.002)^*^ | 0.001 (0.001,0.002)^*^ | 40.5 (27.3,53.2)^*^ |  | 0.002 (0.002,0.005)^*^ | 0.002 (0.001,0.002)^*^ | 0.001 (0.001,0.002)^*^ | 38.3 (27.0,50.9)^*^ |
| Furosemide | 0.004 (0.002,0.007 )^*^ | 0.001 (-0.002,0.003) | 0.003 (0.002,0.006)^*^ | 72.4 (44.0,100)^*^ |  | 0.004 (0.002,0.007)^*^ | 0.002 (-0.002,0.004) | 0.002 (0.001,0.005)^*^ | 59.8 (36.0,100)^*^ |

MBD, metabolic bone disease; LBW, low birth weight.

^a^ Adjusted for maternal demographics.

^b^ Adjusted for maternal demographics, prenatal nutritional conditions, and gestational complications/comorbidities.

^*^A *P* value less than 0.0083 is considered significant after Bonferroni correction.

## Table S6 Associations of comprehensive medication use and the exposure-mediator interaction with MBD incidence: sensitivity analysis 2

| **Effects** | **Model 1^a^** | | |  | **Model 2^b^** | | |
| --- | --- | --- | --- | --- | --- | --- | --- |
|  | **Estimate** | **95% CI** | ***P* value** |  | **Estimate** | **95% CI** | ***P* value** |
| **Prematurity** | | | | | | | |
| Total effect | 0.015 | 0.008–0.071 | 0.040 |  | 0.017 | 0.007–0.069 | 0.040 |
| Direct effect | 0.010 | 0.002–0.101 | 0.040 |  | 0.012 | 0.002–0.097 | 0.040 |
| Indirect effect | 0.005 | -0.030–0.008 | 0.480 |  | 0.005 | -0.030–0.008 | 0.520 |
| Mediation proportion (%) | 36.0 | 4.2–50.4 | 0.520 |  | 27.0 | -46.8–63.9 | 0.560 |
| **LBW** | | | | | | | |
| Total effect | 0.025 | 0.009–0.371 | < 0.001 |  | 0.031 | 0.010–0.347 | < 0.001 |
| Direct effect | 0.011 | 0.006–0.021 | 0.160 |  | 0.016 | -0.002–0.203 | 0.160 |
| Indirect effect | 0.014 | 0.007–0.157 | < 0.001 |  | 0.015 | 0.007–0.141 | < 0.001 |
| Mediation proportion (%) | 54.3 | 41.2–100.0 | < 0.001 |  | 49.4 | 39.6–100.0 | < 0.001 |

MBD, metabolic bone disease; LBW, low birth weight; CI, confidence interval.

A *P* value less than 0.0083 is considered significant after Bonferroni correction.

^a^ Adjusted for maternal demographics.

^b^ Adjusted for maternal demographics, prenatal nutritional conditions, and gestational complications/comorbidities.
